# Comparative metagenomic analysis on COPD and health control samples reveals taxonomic and functional motifs

**DOI:** 10.3389/fmicb.2025.1636322

**Published:** 2025-11-26

**Authors:** Guangyi Chen, Chantal Wiegand, Andreas Willett, Christian Herr, Rolf Müller, Robert Bals, Olga V. Kalinina

**Affiliations:** 1 Graduate School of Computer Science, Saarland University, Saarbrücken, Saarland, Germany; 2 Center for Bioinformatics, Saarland University, Saarbrücken, Germany; 3 Research Group Drug Bioinformatics, Helmholtz Institute for Pharmaceutical Research Saarland (HIPS), Helmholtz Centre for Infection Research (HZI), Saarbrücken, Germany; 4 Department of Internal Medicine V-Pulmonology, Allergology, Infectious Diseases and Critical Care Medicine, Saarland University, Homburg, Germany; 5 Department of Microbial Natural Products, Helmholtz Institute for Pharmaceutical Research Saarland (HIPS), Helmholtz Centre for Infection Research (HZI), Saarbrücken, Germany; 6 PharmaScienceHub, Saarbrücken, Germany; 7 Department of Molecular Therapies for Lung Diseases, Helmholtz Institute for Pharmaceutical Research Saarland (HIPS), Helmholtz Centre for Infection Research (HZI), Saarbrücken, Germany; 8 Medical Faculty, Saarland University, Homburg, Germany

**Keywords:** metagenomics, chronic obstructive pulmonary disease, microbiome, Taxonomic profiling, microbial pathways

## Abstract

Chronic obstructive pulmonary disease (COPD) is a progressive lung condition marked by persistent respiratory symptoms and airflow limitation and significantly affects global health. The intricate relationship between COPD and the lung microbiome has garnered attention, with metagenomic analyses revealing critical insights into microbial community dynamics and their functional roles. In this study, we conducted a comprehensive metagenomic analysis comparing throat samples from COPD patients (*n* = 26) and healthy controls (*n* = 32) derived from a large cohort analyzed at the Saarland University Hospital. Taxonomic profiling and differential abundance analysis indicated a significant reduction of the microbial diversity in COPD patients, with notable overrepresentation of pathogenic bacteria, such as *Veillonella parvula* (NCBI:txid29466), *Streptococcus gordonii* (NCBI:txid1302), *Scardovia wiggsiae* (NCBI:txid230143), as well as a less stable microbiome composition than in healthy individuals. Functional profiling identified alterations in metabolic pathways implicating microbial dysbiosis in disease progression. The study also highlighted enrichment of inflammation-related pathways in COPD samples, emphasizing the microbiome’s role in inflammatory processes. Comparative analysis of bronchoalveolar lavage (BAL) and throat samples collected from the same 11 individuals further underscored distinct microbial compositions across respiratory tract regions, suggesting spatial variability in microbial communities. Metagenomic approaches including analysis of metabolic pathways showed significant alteration of the microbiome of the lung in COPD.

## 1 Introduction

Chronic obstructive pulmonary disease (COPD) is a progressive inflammatory lung disease characterized by persistent airflow limitation and chronic bronchitis or emphysema. It is a leading cause of morbidity and mortality worldwide (3.5 million deaths, fourth most death cases in 2021), significantly impacting the quality of life and placing a considerable burden on healthcare systems (Mayo Clinic, 2020; World Health Organization, 2023). COPD results from long-term exposure to harmful particles or gases, most commonly from smoking, which leads to abnormal inflammatory responses in the lungs (Cleveland Clinic, 2022). The chronic exposure to smoke in COPD causes influx of myeloid cells (macrophages, neutrophils), activation of lymphoid cells, activation of epithelial inflammation and remodeling interaction between inflammatory processes and alterations of the microbiome (O’Donnell et al., 2006). In the COPD-infected samples, changes in the composition and function of the microbiome have been observed. Studies using sputum and bronchoalveolar lavage (BAL) samples have shown distinct microbial communities in the upper and lower respiratory tracts of COPD patients (Zakharkina et al., 2013). Recent 16S rRNA gene sequencing and shotgun/metagenomic studies demonstrate that these variations may associate with disease status, severity, and exacerbation risk and influence disease progression and exacerbation frequency (Ramirez et al., 2021; Pathak et al., 2020; Tangedal et al., 2024).

Metagenomic sequencing offers a culture-independent approach that enables comprehensive profiling of the microbial communities and their functional potentials directly from clinical samples (Pérez-Cobas et al., 2020). Metagenomic profiling involves the extraction and sequencing of microbial DNA from clinical samples, followed by bioinformatics analysis to identify microbial taxa (taxonomic profiling) and their functional genes and pathways (functional profiling) (Aguiar-Pulido et al., 2016). This approach allows for high-resolution analysis of the compositional microbiome, providing insights into the potential roles of specific microbes and their metabolic pathways in COPD, and further uncovers alterations in metabolic pathways related to lipid metabolism, oxidative stress, and immune responses in COPD patients (Bowerman et al., 2020; Dora et al., 2024).

Differential abundance analysis (DAA) is a critical component of metagenomic studies, as it identifies microbial taxa and functional genes/pathways that are significantly associated with disease states (Yang and Chen, 2022). To provide a more robust perspective, these findings are typically complemented by multivariate community-level analyses (e.g., ordination and PERMANOVA), which demonstrate overall differences in microbial composition between groups and thereby strengthen the evidence for disease-associated shifts (Kleine Bardenhorst et al., 2021; Xia and Sun, 2017). In the context of COPD, such analyses have highlighted specific bacterial species and functional pathways that are differentially abundant in patients compared to healthy controls. For instance, the increased presence of *Proteobacteria* and the depletion of beneficial commensals like *Firmicutes* have been linked to disease severity and exacerbations. In healthy individuals, the predominant phyla in health lungs are *Firmicutes* and *Bacteroidetes*, followed by Proteobacteria and Actinobacteria (Hou et al., 2022). Altered abundance of *Pseudomonas*, *Moraxella*, *Lactobacillus*, and *Haemophilus* have been identified during COPD exacerbations (Millares et al., 2014). The airway microbiome of COPD patients is typically characterized by a reduction in microbial diversity and an overrepresentation of potentially pathogenic bacteria in genera such as *Streptococcus*, *Pseudomonas*, *Moraxella* and *Haemophilus* using 16S rRNA gene amplification (Ramsheh et al., 2021; Millares et al., 2014). These alterations can disrupt the homeostasis of the respiratory tract, leading to increased inflammation and exacerbations (Po et al., 2011; De Matteis et al., 2019). Another type of analysis focuses on differentially represented genes and pathways. Specifically, COPD patients exhibited an enrichment of genes related to virulence, antibiotic resistance, and inflammation (Kayongo et al., 2022).

The aim of this study was to perform a detailed comparison of the microbiomes from upper respiratory tract samples from COPD patients and healthy controls from the IMAGINE study (Schmartz et al., 2024), as well as BAL samples from the University Hospital Saarland, applying metagenomic analysis of taxonomic and functional profiling. We demonstrate significant differences in the diversity and composition of the microbiome between COPD patients and controls already in the throat samples, alleviating the need to obtain sputum samples. We highlight inflammation-related genes and pathways that are enriched in the samples from the COPD patients.

## 2 Materials and methods

### 2.1 Sample collection and study design

This study capitalizes on the data collected by the IMAGINE consortium (Schmartz et al., 2024). The whole IMAGINE cohort consists of 3,483 samples from 657 individuals spanning different body sites including saliva, interdental plaque, conjunctival swabs, throat swabs, stool, skin swabs, and so on. The disease information of these 657 patients was also documented. To focus on the respiratory system, we selected the available throat samples from 32 normal health control individuals (without any disease) and 26 COPD patients, forming the two groups for this study (Supplementary Table 1). Additionally, in order to draw the comparison between bronchoalveolar lavage (BAL) and throat samples, we selected the 11 individuals whose BAL (acquired from the University Hospital Saarland) and throat samples are both available. Among them 3 individuals are COPD patients overlapped with the COPD patients in comparison 1, the rest 8 are other non-health individual (Supplementary Table 1). These individuals are all from the IMAGINE study (Schmartz et al., 2024). The BAL samples are internally collected from the University Hospital Saarland and not a part of the IMAGINE study.

**Figure 1:**
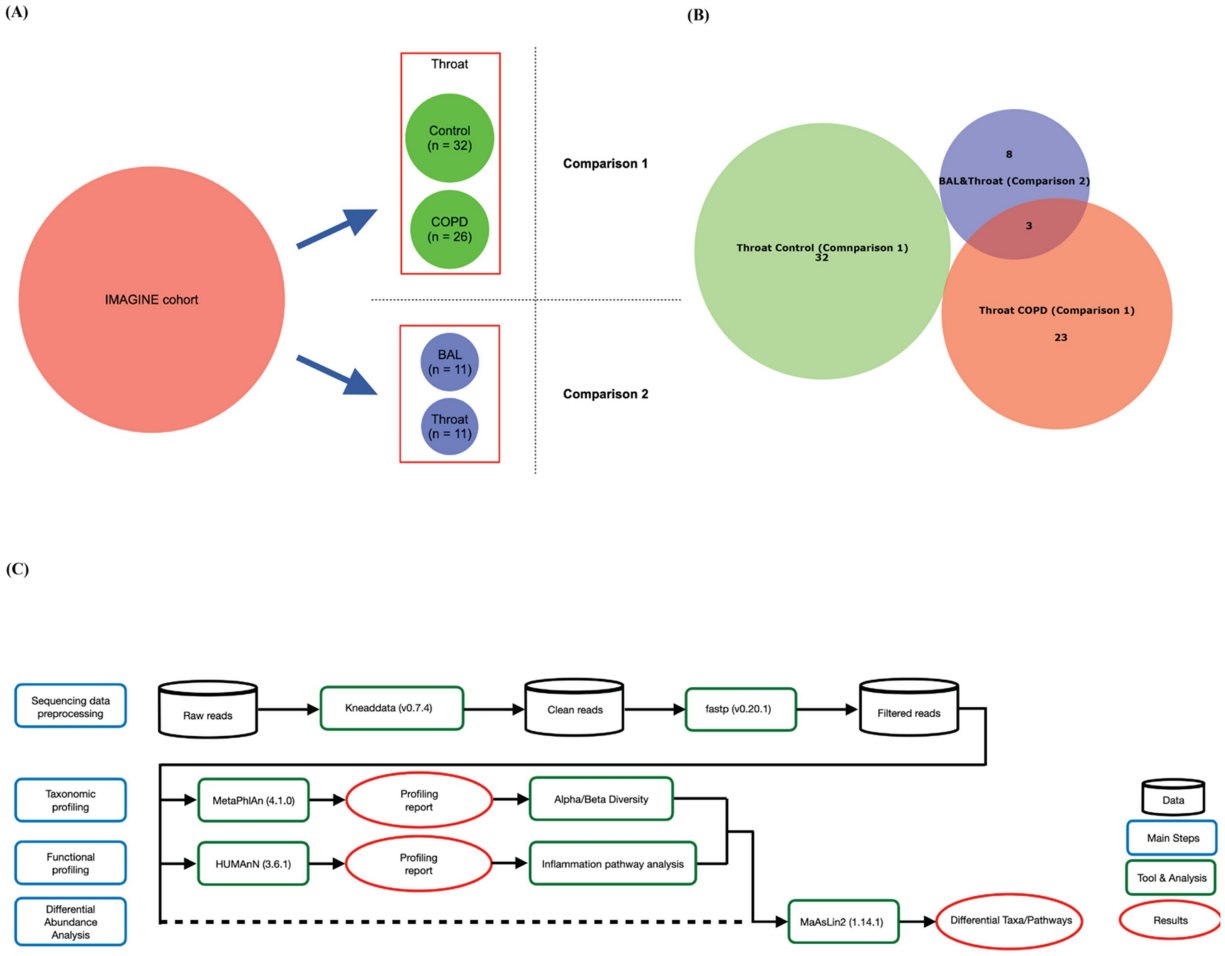
Design and Bioinformatics workflow of this study. **(A)** Study design. A total of 1931 high-quality metagenomic samples were obtained from IMAGINE cohort. Two sets of samples were compared in this study: Comparison 1 is used to compare the taxonomic and functional profiles of the COPD and control groups. Comparison 2 focused on comparing the taxonomic profiles between BAL (acquired additionally from the University Hospital Saarland) and throat samples collected from the same individuals. **(B)** Venn diagram of selected individuals in this study. **(C)** Workflow of this study.

We designed two comparisons (Figure 1A): comparison 1 focuses on testing the taxonomical and functional differences between the throat samples in COPD and control groups; comparison 2 is designed to test if there are significant differences in terms of the microbiome compositions between the BAL and throat samples (Figure 1B).

### 2.2 Metagenomics analysis

The computational pipeline of this study is shown in Figure 1C.

For all the throat samples, we downloaded the preprocessed reads directly from the IMAGINE study in Sequencing Read Archive (SRA) under the accession code PRJNA1057503. For all the BAL samples, we collected the processed reads internally and uploaded to SRA under the accession code PRJNA1327646. All the processed reads were applied uniformly with the following pipeline from the IMAGINE study: The raw paired-end reads were firstly processed with Kneaddata (v0.7.4) to remove human reads contamination (Beghini et al., 2021). The clean reads were fed into fastp (v0.20.1) to trim out the low-quality reads (Chen, 2023). MultiQC (v1.11) was used to visualize the results (Ewels et al., 2016). The remaining filtered reads were used for further analysis.

To perform the taxonomic profiling, MetaPhlAn4 (4.1.0) was run on the filtered reads of each sample using the reference database mpa_vJun23_CHOCOPhlAnSGB_202307 to get the profiling report for each sample (Blanco-Míguez et al., 2023). The relative counts were normalized to 100%. The individual samples were merged into an aggregated text file. These profiling reports were used for further calculation of alpha and beta diversity using the auxiliary utilities from the same tool. Species and genus-level abundances were extracted for visualization (using hclust2 v1.0.0; SegataLab, 2020) and further differential abundance analysis.

For functional profiling, HUMAnN3 (3.6.1) with nucleotide database full_chocophlan_v201901_v31, translation database UniRef90, and the taxonomic profile from the previous step for each sample was employed (Beghini et al., 2021; Suzek et al., 2015). The output from this tool, namely identified MetaCyc pathway abundances with contributions from each specific species (stratified outputs), were then normalized to relative abundances, and individual samples were merged into an aggregated text file. In MetaCyc, microbial pathways are defined as metabolic pathways or biochemical reaction networks that are found in microbes (e.g., bacteria, archaea, fungi). MetaCyc provides detailed information about these pathways, describing how specific sequences of enzymatic reactions transform substrates into products (Caspi et al., 2020). We extracted the total abundance (unstratified) for each pathway from the aggregated profiles for further differential abundance analysis. In order to investigate the dynamics of pathways that are involved in inflammation, we searched the pathways that are relevant to inflammation in the MetaCyc database through a literature review and mapped them back to the pathway abundance results. Pathways were visualized using the ‘Pathway Collages’ tool from the MetaCyc website.

Welch’s *t*-test and Chi-square test were performed using Python package scipy (v 1.16.2). PERMANOVA and Principal Coordinate Analysis (PCoA) analysis were performed using Python package scikit-bio (v0.7.0). We used the R package MaAsLin2 (version 1.14.1) to perform differential abundance analysis, fitting generalized linear models to identify microbial features significantly associated with the primary grouping factor (Comparison 1: COPD vs. Control; Comparison 2: Throat vs. BAL). For the comparison of throat samples between the COPD and control groups (Comparison 1), we focus on testing the microbial features of taxonomic profiles, functional profiles, and alpha diversity, by applying the following test settings: (taxonomic profiles and functional profiles) fixed effects: group (COPD and control, main interest for testing), age, sex and BMI (covariates); analysis method: linear model; the minimal required prevalence: 10%; Benjamini-Hochberg correction; the Total Sum Scaling (TSS) normalization; log transformation (Mallick et al., 2021). For taxonomic profiles, we focused on testing the species and genus-level relative abundances. For functional profiles, we focused on testing the unstratified profile (community-level abundance) to reduce the number of features (taxonomic profiles and functional profiles). For the comparison between BAL and throat samples (Comparison 2), we focus on testing the microbial features of the alpha diversity by applying the following test settings: fixed effect: group (COPD and control, main interest for testing), age, sex, and BMI (covariates); analysis method: linear model; the minimal required prevalence: 10%. We tested for alpha diversity indices in Shannon and Simpson metrics (Shannon, 1948; Simpson, 1949). Venn diagrams used in this study were created using DeepVenn (Hulsen, 2022).

## 3 Results

### 3.1 Summary of study participants

In the IMAGINE cohort, each individual is associated with metadata including information on, for example, disease status, age, sex, etc. We identified 38 COPD patients and 46 healthy individuals (participants without any known disease) as the healthy controls in this cohort. To focus on COPD-relevant probes, we selected throat samples, resulting in 32 and 26 available throat samples for the COPD and control groups, respectively (Table 1). The age and BMI between the COPD and control group are significantly different (Welch’s t-test, age: *p* = 0.0000; BMI: *p* = 0.0164). Additionally, to compare the microbiome composition between BAL and throat samples, we collected data for 11 individuals (among which 3 are COPD patients, Figure 1B), whose BAL and throat samples are both available (Table 1). See Supplementary 1 for the complete metadata.

**Table 1:** Baseline summary of the individuals in this study.

Comparison 1		
	Control (*n* = 32)	COPD (*n* = 26)
Age*	24.34 ± 3.20	65.35 ± 8.18
Sex (Male/Female)	15/17	16/10
BMI (kg/m2)*	22.90 ± 3.39	26.58 ± 6.65

Comparison 2 (*n* = 11)	
Age	63.6 ± 9.25
Sex (Male/Female)	8/3
BMI (kg/m2)	24.45 ± 4.01

### 3.2 Both taxonomic and functional profiles show a higher diversity in the control group over the COPD group

**Figure 2:**
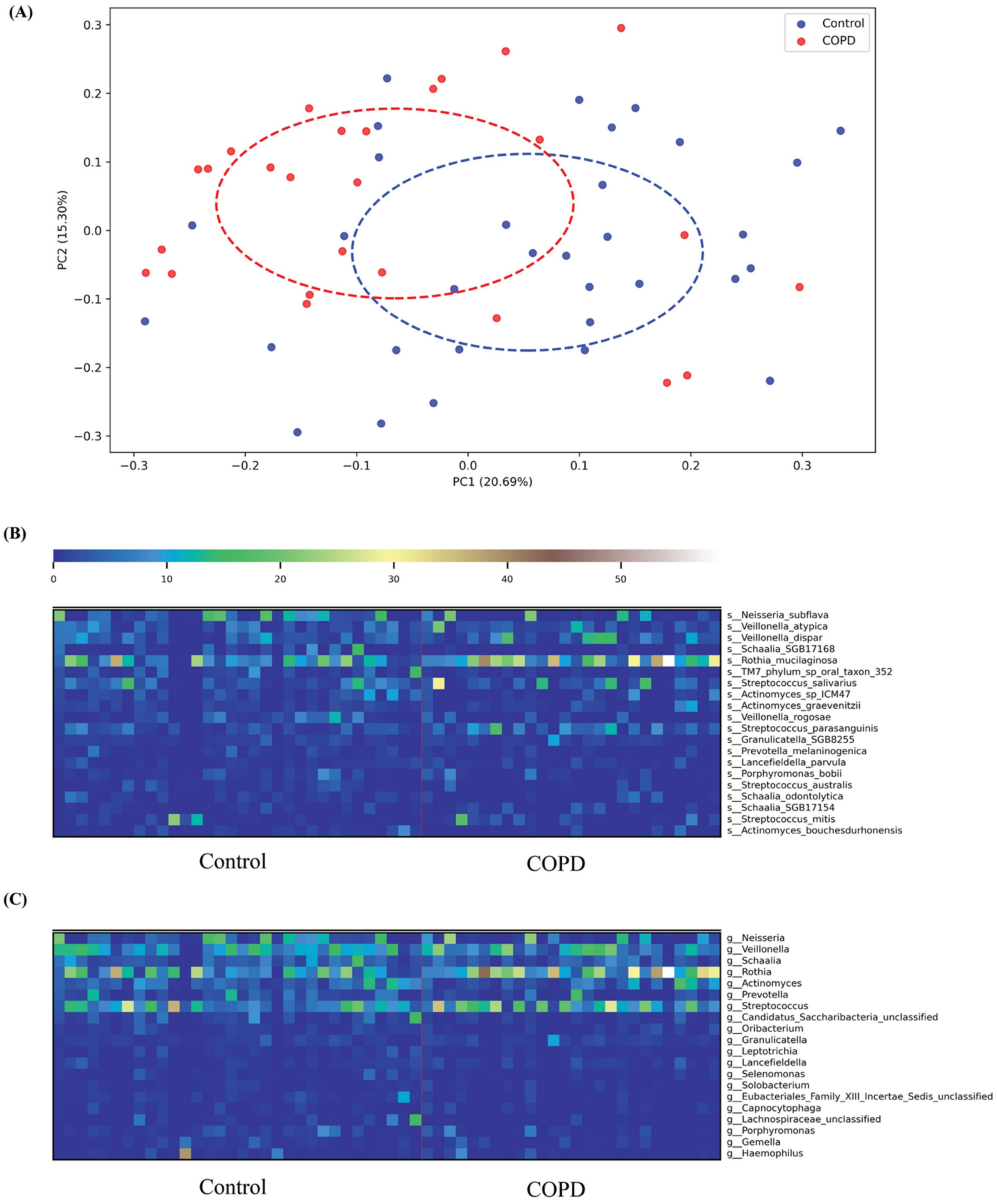
COPD and control species profiling comparison. **(A)** Principal Coordinates Analysis (PCoA) based on Bray–Curtis dissimilarity of throat microbiome samples from COPD patients and healthy controls. Each point represents a sample, colored by group (blue: Control; red: COPD). Dashed ellipses indicate the 95% confidence interval of each group, showing partial separation along the first two principal coordinates (PC1: 20.69% variance explained; PC2: 15.30% variance explained). **(B)** The top 20 variable species in both the COPD and control groups; **(C)** The top 20 variable genera in both the COPD and control groups.

PERMANOVA analysis indicated a statistically significant difference in throat microbiome composition at the species level between COPD and control cohorts (Bray–Curtis dissimilarity; pseudo-*F* = 3.16, *p* = 0.002, 999 permutations). This result was corroborated by the principal coordinates analysis (PCoA), which revealed clear group separation along the first two principal coordinates based on Bray–Curtis dissimilarities (Figure 2A).

Taxonomic profiling results have shown that more different bacterial species and genera have been detected in the control group than in the COPD samples (control: 230, COPD: 179, shared: 151). For the control group, we found that the most abundant species detected include *Neisseria subflava* (NCBI:txid28449), *Rothia muciladinosa* (NCBI:txid43675), *Veillonella dispar* (NCBI:txid39778), *Veillonella atypical* (NCBI:txid39777), and *Schaalia* species (NCBI:txid2529408) and the most abundant genera are *Neisseria* (NCBI:txid482), *Veillonella* (NCBI:txid29465), *Schaalia*, *Rothia* (NCBI:txid32207), and *Actinomyces* (NCBI:txid1654), the results are largely consistent with the findings reported in the previous study (Natalini et al., 2023). For the COPD group, we detected similar species and genera as most abundant, but their distribution is skewed compared to the control group, with a more dominant abundance for *Rothia mucilaginosa* on the species level and *Veillonella* on the genus level (Supplementary Figures 1A,B).

**Figure 3:**
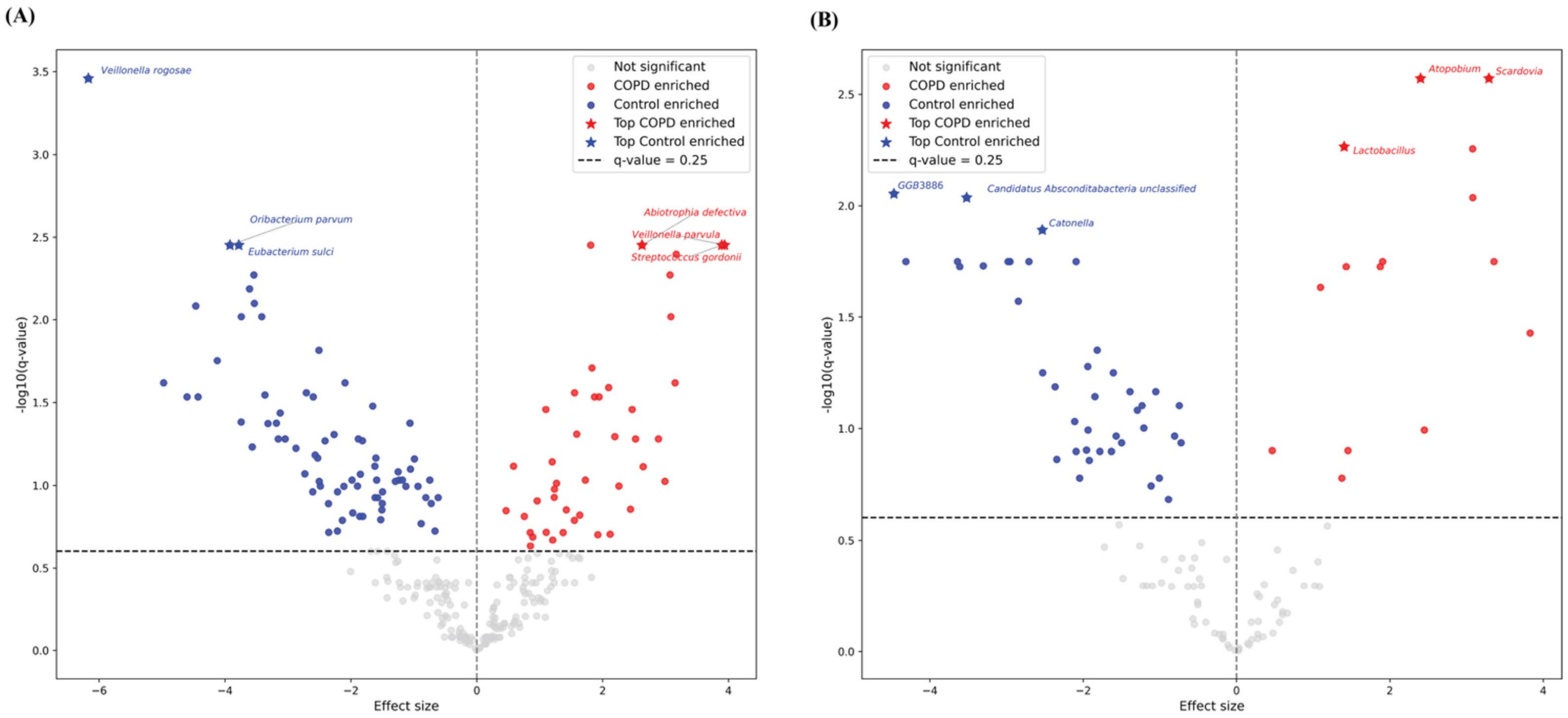
Volcano plots of differential abundant taxa between COPD and control groups in throat samples using MaAsLin2. Each point represents one taxon, plotted by effect size (*x*-axis) and –log10-transformed q-value (y-axis). Gray points indicate non-significant species, while red and blue points denote COPD- and control-enriched species, respectively. Stars highlight the top three significantly enriched taxa per group. The horizontal dashed line marks the default significance threshold at *q*-value = 0.25 by MaAsLin2. **(A)** Results for species-level. **(B)** Results for genus-level.

Among the top 20 species and genera with the largest abundance variation across all COPD and control samples (Figures 2B,C), we observed that the most variable taxa for healthy controls agree well with the those observed in the sample-wise profiles, while the COPD samples have higher variable abundances for these taxa. Further, differential abundance analysis revealed 73 species and 40 genera significantly enriched in the control group, and 43 species and 15 genera significantly enriched in the COPD group (Supplementary Tables 2, 3). The results align closely with the findings of the previous study (Natalini et al., 2023) (Figure 3).

**Figure 4:**
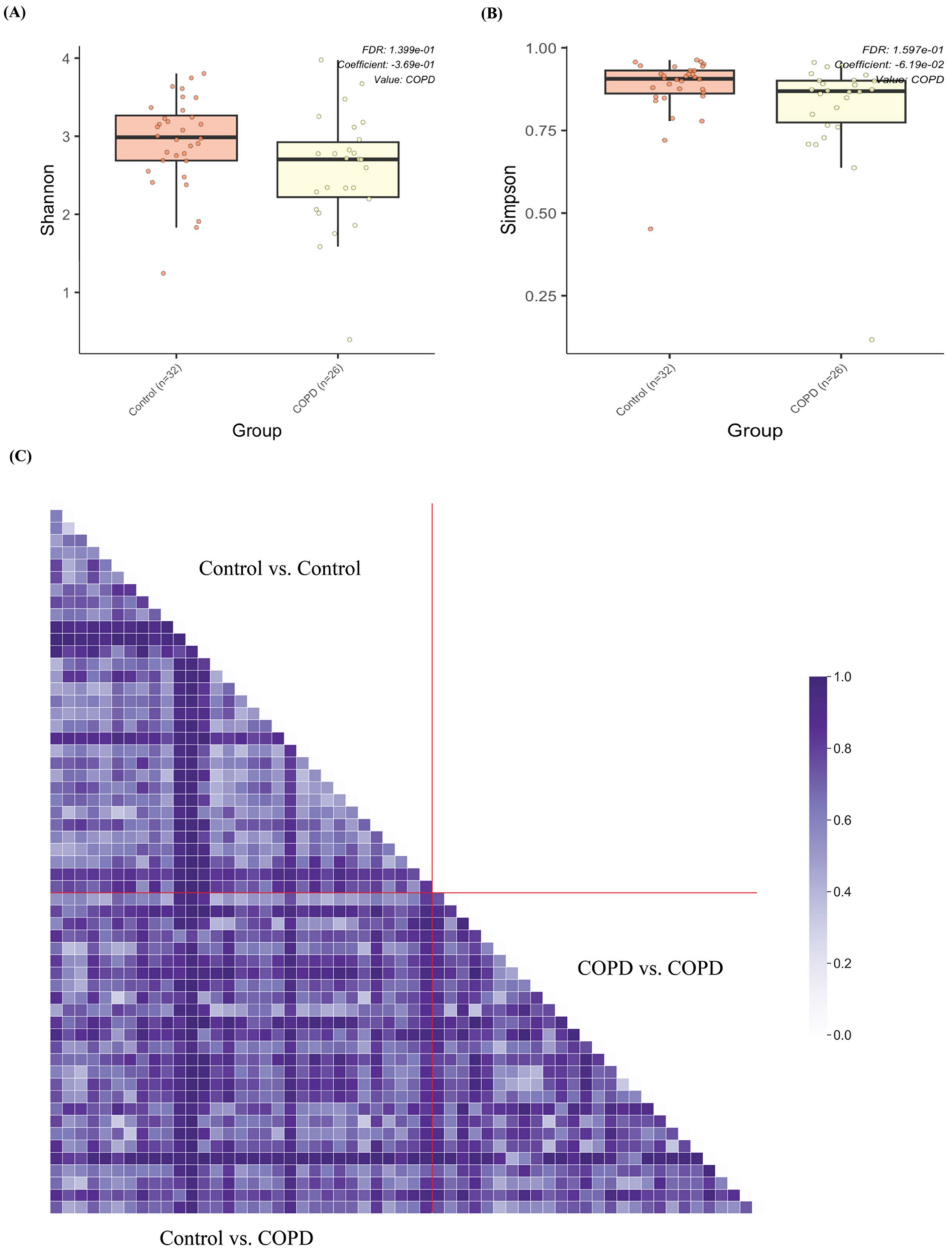
Alpha and beta diversity of samples between the COPD and control group. **(A)** Box plot of alpha diversities in Shannon metrics show statistically significant differences between the COPD and control groups. **(B)** Box plot of alpha diversities in Simpson metrics show statistically significant differences between the COPD and control groups. **(C)** Grouped pairwise beta diversities (Bray-Curtis) in intra-group comparisons (control vs. control, COPD vs. COPD) and inter-group comparison (COPD vs. control).

Interestingly, the control samples have statistically significantly higher alpha diversities (Shannon and Simpson) than the COPD samples (Figures 4A,B and Supplementary Figure 2). Beta diversity (Bray-Curtis) analysis indicated that the control group showed slightly smaller inter-group diversity (0.658 ± 0.193) compared to the COPD group (0.726 ± 0.197) (Figure 4C). These results suggest that the lung microbiome of COPD patients tends, on one hand, to comprise fewer different bacteria, but on the other hand, has a more variable composition between patients, as compared to the healthy controls.

**Figure 5:**
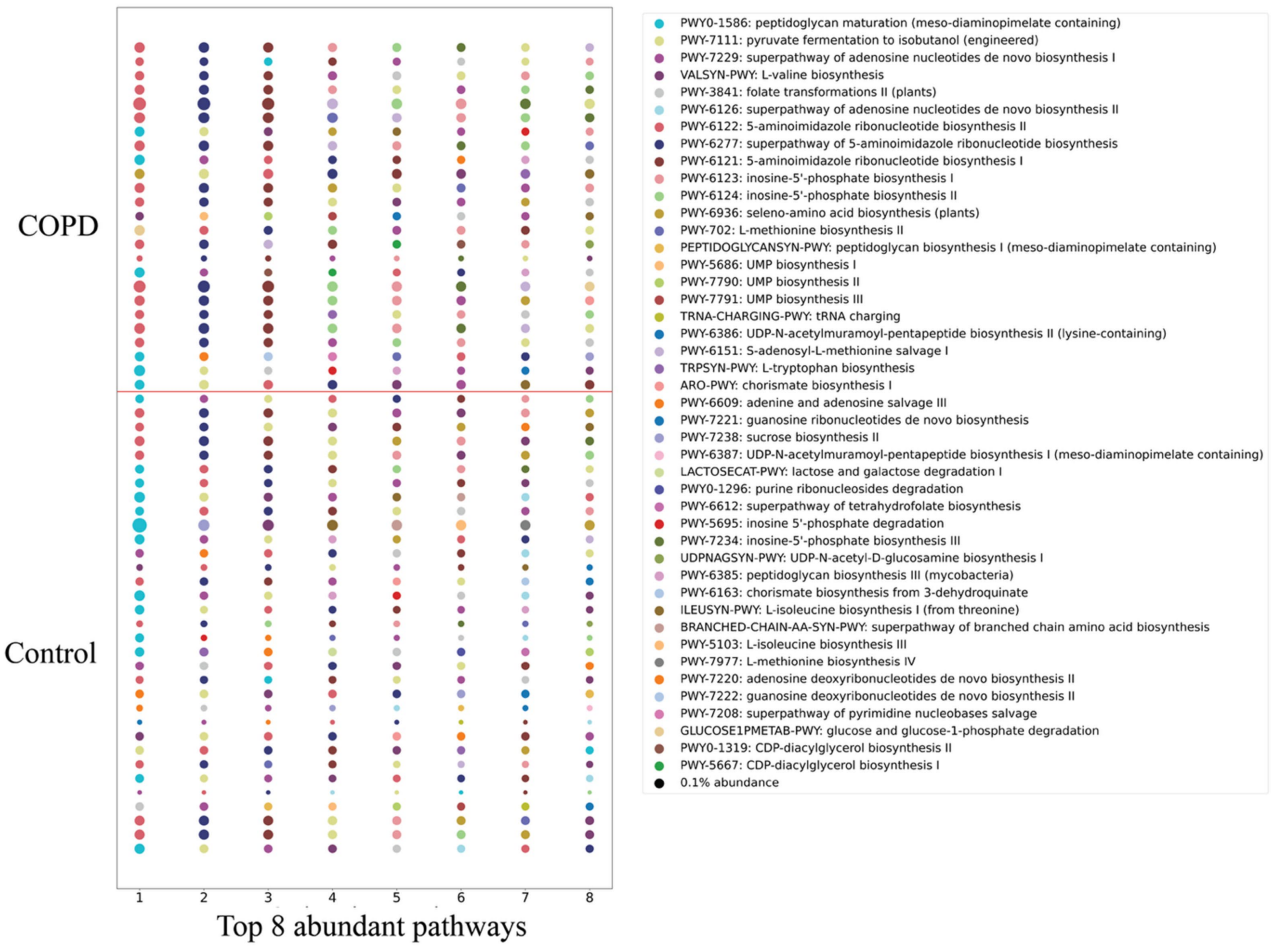
Comparison of detected pathways between the COPD and control group. The top 8 abundant pathways in each sample of the COPD and control groups. Circle size is proportional to the relative abundance.

Functional profiling identified a total of 430 microbial pathways (metabolic pathways or biochemical reaction networks that are found in microbes, e.g., bacteria, archaea, fungi) across all the samples, where 382 pathways are shared between the COPD and control groups. The control group contains a higher number of pathways than the COPD group. The abundance of each pathway was determined by summing the abundances of its constituent reactions, inferred from gene family abundances mapped to enzymatic functions, and adjusted for pathway completeness and sequencing depth. By analyzing the eight most abundant pathways per sample (Figure 5), we found that the control group exhibits a greater number of distinctive abundant pathways in total than the COPD group (control: 38; COPD: 32). Differential abundance analysis suggests that 21 pathways are significantly enriched in the control group, and 55 pathways are significantly enriched in the COPD group (Supplementary Table 4).

### 3.3 Inflammation-related pathway enrichment analysis

**Figure 6:**
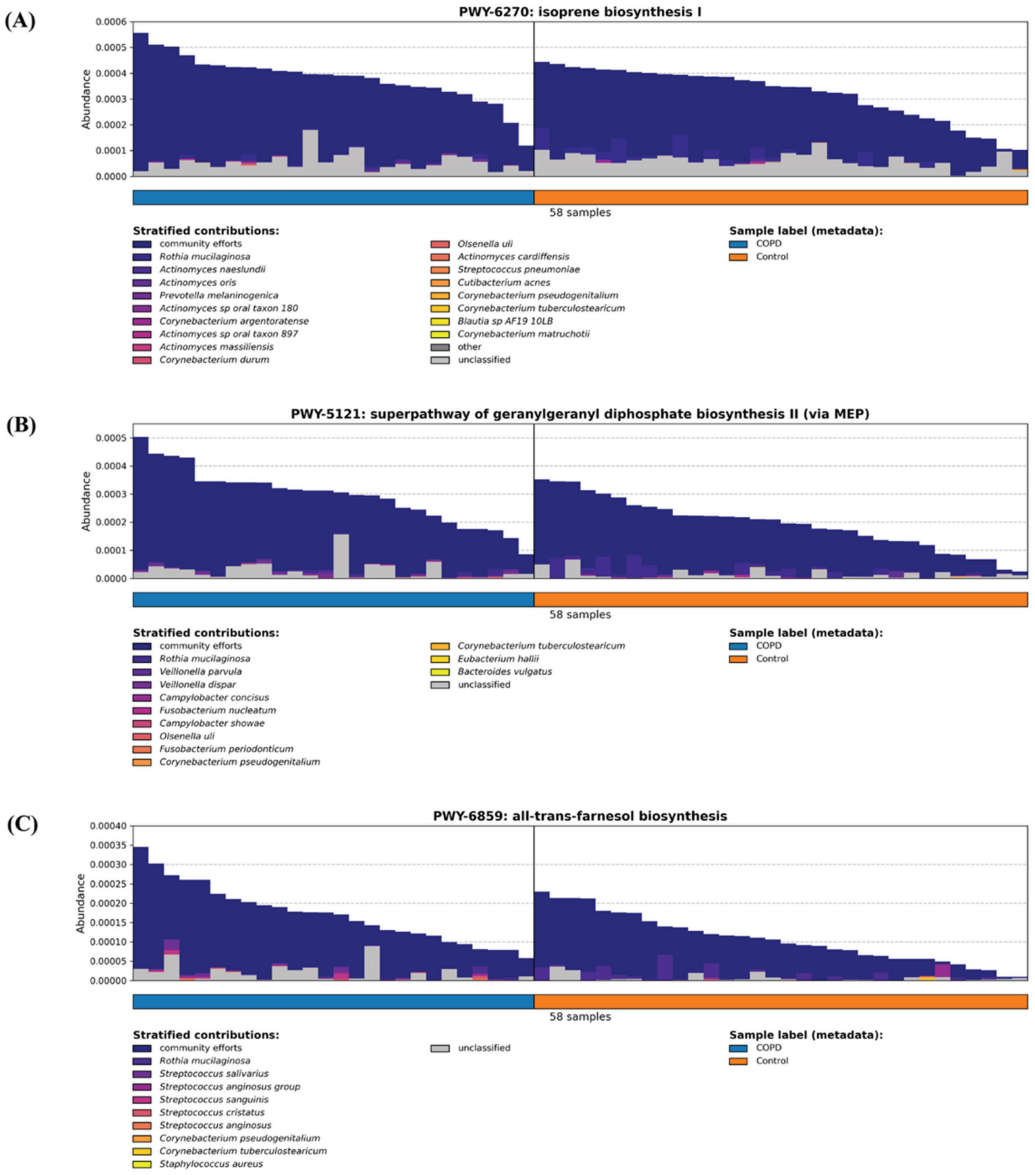
Three inflammation-related pathways **(A)** isoprene biosynthesis I (via MEP) (BioCyc Id: PWY-6270), **(B)** superpathway of geranylgeranyl diphosphate biosynthesis II (via MEP) (BioCyc Id: PWY-5121), and **(C)** all-*trans*-farnesol biosynthesis (BioCyc Id: PWY-6859) are enriched in the COPD samples.

Previous studies have demonstrated that isoprenoids, in particular farnesyl pyrophosphate (FPP), geranylgeranyl giphosphate (GGPP) and farnesol, play a key role in inflammation response (Marcuzzi et al., 2008; Santoro et al., 2018) (Supplementary Figure 3). Between our COPD and control groups, the COPD are enriched in three pathways assisting isoprenoid production: isoprene biosynthesis I (via MEP) (BioCyc Id: PWY-6270), superpathway of geranylgeranyl diphosphate biosynthesis II (via MEP) (BioCyc Id: PWY-5121) and all-*trans*-farnesol biosynthesis (BioCyc Id: PWY-6859). By examining the stratified contributors to each pathway, we cannot identify a single major contributing species (where they come from), but rather we observe a community effort from various bacteria across different samples, possibly caused by the infection stimulating the joint proliferation of bacteria harboring these pathways (Figure 6).

### 3.4 BAL samples show significant difference with throat samples

**Figure 7:**
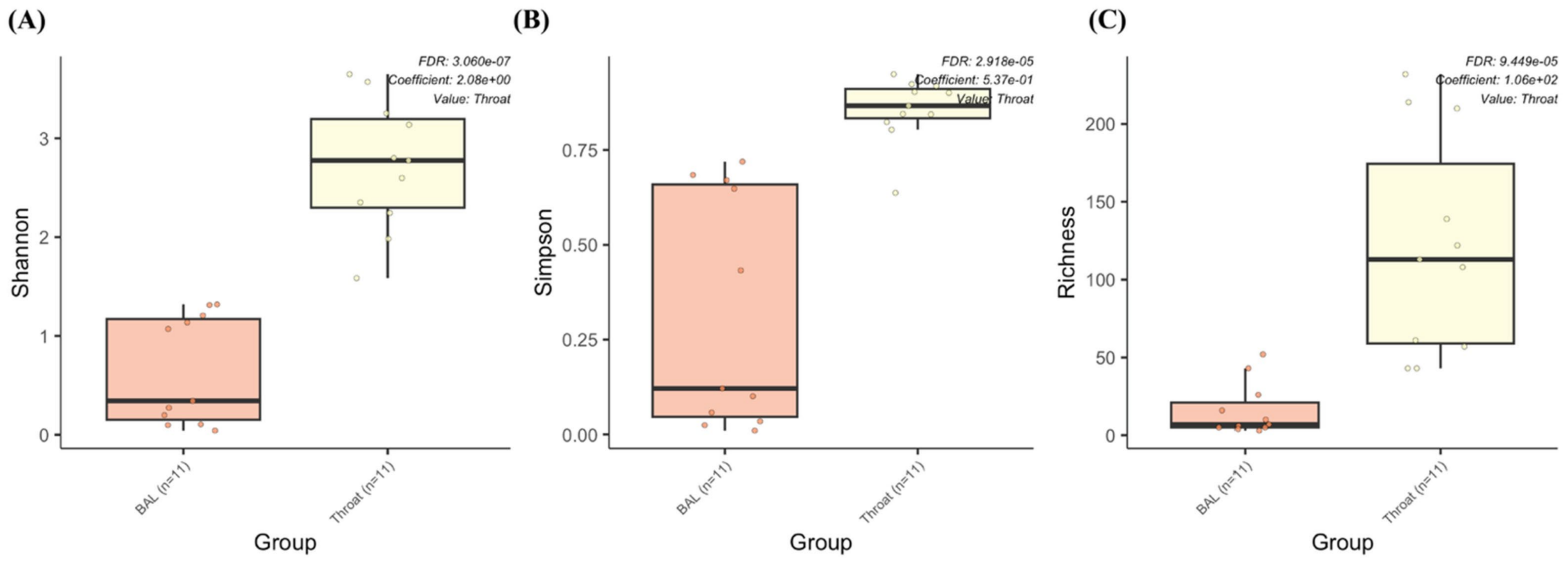
The microbiome abundance comparison between BAL and throat samples. Box plot of alpha diversities in **(A)** Shannon, **(B)** Simpson and **(C)** Richness metrics show statistically significant differences between the throat and BAL groups.

We also performed a comparison between the BAL samples and throat samples from 11 participants to evaluate whether pharyngeal samples can replace BAL samples for metagenomic and metabolomic analysis, since BAL samples depend on an invasive procedure of bronchoscopy. However, the throat samples contain more species (throat: 392; BAL: 82; shared: 62) and genera (throat: 166; BAL: 59; shared: 44) than the BAL samples. Differential abundance analysis also shows significantly higher alpha diversities (Shannon, Simpson, and richness) from the throat samples (Figure 7), which makes it difficult to replace one with the other.

## 4 Discussion

In this study, we compared upper respiratory tract microbiomes of COPD patients and healthy individuals. We conclude, first, that the control group exhibits greater taxonomic and functional diversity compared to the COPD group; second, that in the COPD group, three pathways involved in isoprenoid production are enriched, which supports the notion of the inflammatory response in COPD; and third, that bronchoalveolar lavage (BAL) samples differ significantly from throat samples.

COPD is a complex disease whose mechanisms are not yet fully understood. It involves interactions among bacteria within the human lung microbiome environment. To understand the disease mechanisms, it is essential to understand the role of microbiomes and its functional capabilities. Thanks to the recent development of sequencing technology and metagenomics methods, we are now in a position to gain a better understanding of that. In recent years, several studies have leveraged metagenomic approaches to explore the microbial and functional landscape of COPD. High-throughput sequencing has been used to analyze the lung microbiomes of COPD patients, identifying significant alterations in microbial diversity and functional genes related to inflammation and immune response (Cameron et al., 2016). Another study focused on the microbiome diversity in the bronchial tracts of COPD patients using high-throughput sequencing, revealing that COPD patients have a significantly different microbial composition compared to healthy individuals (Cabrera-Rubio et al., 2012). Furthermore, a comprehensive study analyzed sputum samples from COPD patients and controls and identified biomarkers that are significantly elevated in COPD patients. These biomarkers are associated with disease severity and can predict future exacerbations, implicating pathways such as mucus hydration, adenosine metabolism, and oxidative stress as potential therapeutic targets (Esther et al., 2022). All these findings agree well with the results of the study presented here. Despite these advancements, limitations persist. Regardless of comprehensive metagenomic studies on microbial organisms, genes, and pathways, they do not always clarify which microbial species are actively contributing to disease pathology. Functional metagenomics is still in its infancy, and interpreting the vast amount of data generated remains a significant challenge. Future research should focus on integrating multi-omics approaches and longitudinal studies to better understand the dynamic interactions between the lung microbiome and COPD pathogenesis.

This study contributes to the progress on the field in several aspects. First, the analysis of the taxonomic and functional profiles of the COPD and control groups throat samples and of the microbiomes contributed to the understanding of the species diversity and its change in the disease. Second, a systematical comparison of the COPD and control groups indicates that the microbiomes and pathways that are significantly different. Third, the characterization of the pathways involving the inflammation process and of other inflammation-related pathways demonstrates that they are enriched in the COPD samples. The detected microbiomes in COPD samples from our study align closely with those reported in the previous research (Cameron et al., 2016; Cabrera-Rubio et al., 2012; Wang et al., 2021). Furthermore, our findings on higher alpha diversity in the control group over the COPD group are consistent with the previous study (Diao et al., 2017). Additionally, we identified a higher beta diversity in the COPD samples, which, together with our observations on alpha diversity, indicate that microbiome in COPD patients is narrower and destabilized. This finding aligns well with the prior research and further strengthens our comprehension of the microbiome community within the intricate landscape of COPD (Sin, 2023). A key innovation of our study lies in its comprehensive functional profiling of samples, particularly the comparison of inflammation-related pathways between COPD and control groups. This contrasts with previous studies that predominantly focused on other pathways, such as bacterial growth, or focused on the mechanisms of the inflammation-related pathway itself (Cameron et al., 2016; Yamada and Ichinose, 2018). Earlier studies have shown that isoprenoids such as farnesyl pyrophosphate (FPP), geranylgeranyl diphosphate (GGPP), and farnesol play central roles in regulating inflammation and immune signaling (Marcuzzi et al., 2008; Santoro et al., 2018). The enrichment of three isoprenoid-related microbial pathways in COPD-associated microbiomes (isoprene biosynthesis I (via MEP) (BioCyc Id: PWY-6270), superpathway of geranylgeranyl diphosphate biosynthesis II (via MEP) (BioCyc Id: PWY-5121) and all-trans-farnesol biosynthesis (BioCyc Id: PWY-6859)) identified by this study suggests a clinically relevant metabolic link between microbial activity and chronic airway inflammation. Specifically, the microbial pathway of isoprene biosynthesis leads to the formation of precursors for nonsterol isoprenoids such as farnesyl and geranylgeranyl derivatives that play essential roles in immune regulation and inflammation control (Houten et al., 2003). Further, farnesol biosynthesis can downregulate the expression of inflammatory mediators and act as a virulence factor by inducing anti-inflammatory responses and suppressing pro-inflammatory cytokines, thereby increasing host susceptibility to infection (Jung et al., 2018). Together, these findings point to microbial isoprenoid metabolism as a clinically relevant contributor to airway inflammation in COPD and a potential target for therapeutic modulation.

We also compared samples from BAL with those from pharyngeal swabs to evaluate whether both sample types correlate and established a significantly lower microbiome diversity in the BAL samples compared to the pharyngeal swabs. This conclusion aligns with the well-established ecological split between the upper and lower airways. Oropharyngeal communities are consistently more diverse and cluster separately from lung communities, reflecting the upper airway’s higher biomass and frequent immigration from the oral cavity. In contrast, the lower airways are a low-biomass environment shaped by stronger niche filtering and host defenses. Previous study has also validated this conclusion and reported greater diversity in oropharyngeal/throat swabs than in BAL, with clear community separation (Kirst et al., 2019). Clinically, reduced *α*-diversity in the lower airways is often interpreted as a shift toward dysbiosis or domination by a few taxa, which may compromise ecological resilience and cost the lung susceptible to pathogen overgrowth or inflammation (Dickson et al., 2020). In chronic airway disease such as COPD, lower airway microbiome alterations and loss of diversity have been associated with more frequent exacerbations and adverse clinical trajectories (Li et al., 2024). The diminished diversity in BAL relative to throat samples underscores the possibility that changes in the lower-airway microbiota may more closely reflect disease processes or prognostic risk than do surrogate upper-airway samples (Mikhail and O’Dwyer, 2025).

In conclusion, this study provides important contributions to our understanding of the COPD-associated microbiome and its functional capabilities. The insights gained could trigger future efforts to identify microbiome-based biomarkers or therapeutic targets, ultimately aiding in the development of more personalized and effective treatment strategies for COPD.

## Data Availability

The throat samples metagenomic sequencing data after removing ambient human DNA analyzed for this study was retrieved from the Sequencing Read Archive under the accession code PRJNA1057503 (https://www.ncbi.nlm.nih.gov/bioproject/PRJNA1057503). The BAL samples metagenomic sequencing data after removing ambient human DNA analyzed for this study has been deposited in the Sequencing Read Archive under the accession code PRJNA1327646 (https://www.ncbi.nlm.nih.gov/bioproject/PRJNA1327646). Details of the sample sources are provided in Supplementary Table 5.
