# Supplementary material for: Comparative metagenomic analysis on COPD and health control samples reveals taxonomic and functional motifs
Source: Front Microbiol. 2025 Nov 26;16:1636322. doi: 10.3389/fmicb.2025.1636322 (PMC12689518; doi:10.3389/fmicb.2025.1636322)
Supplement: Supplementary file 1 [file Supplementary_file_1.docx]

Supplementary Figures


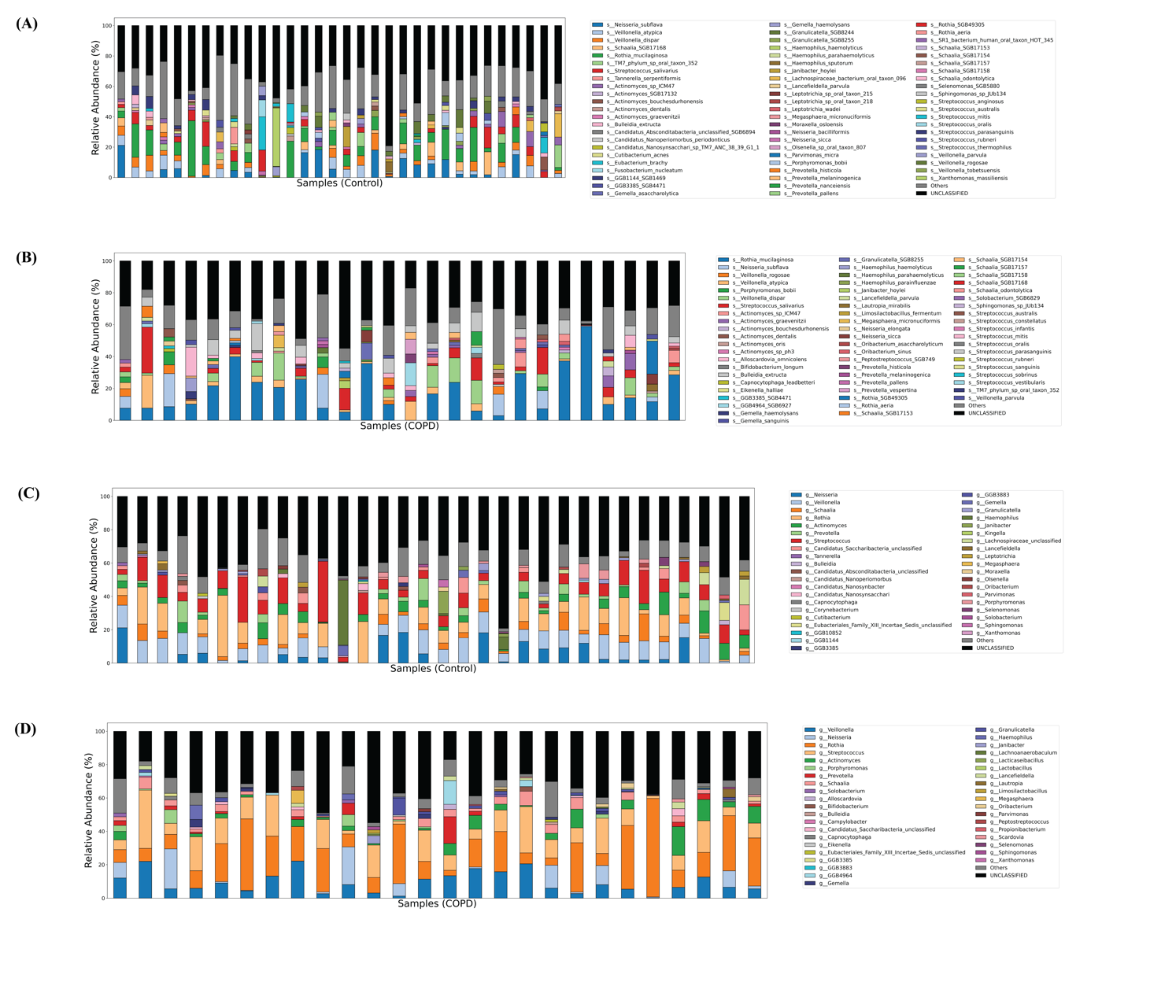


**Supplementary Figure 1.** Control and COPD species profiling comparison. (A). Species-level taxonomic profiles of throat samples in the control group; (B). Species-level taxonomic profiles of throat samples in the COPD group; (C). Genus-level taxonomic profiles of throat samples in the control group; (D). Genus-level taxonomic profiles of throat samples in the COPD group. The top 10 abundant taxa for each sample were shown, the others were marked in the 'Others' category.


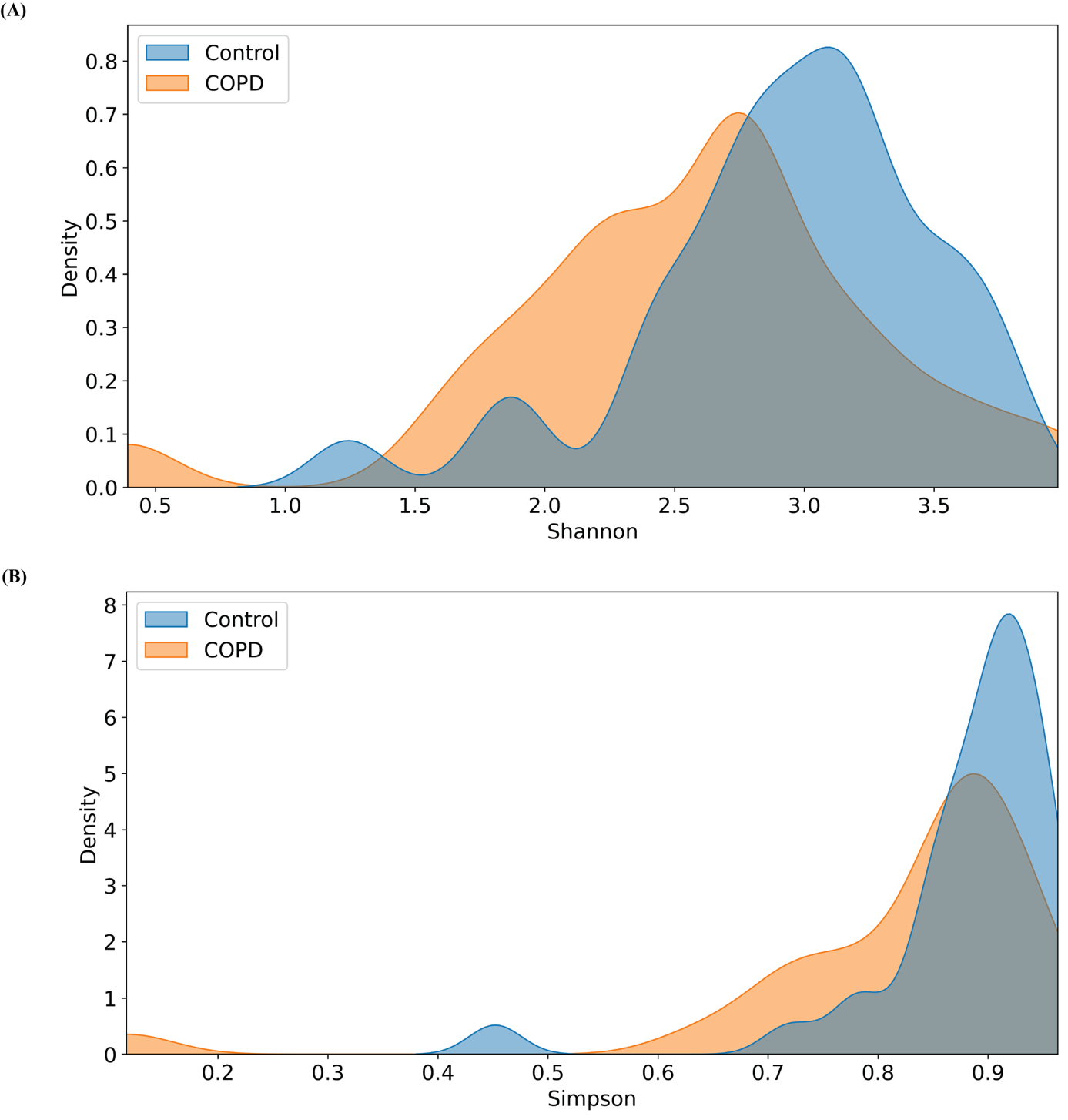


**Supplementary Figure 2.** Box plot of alpha diversities in (A) Shannon and (B) Simpson metrics between the COPD and control groups.


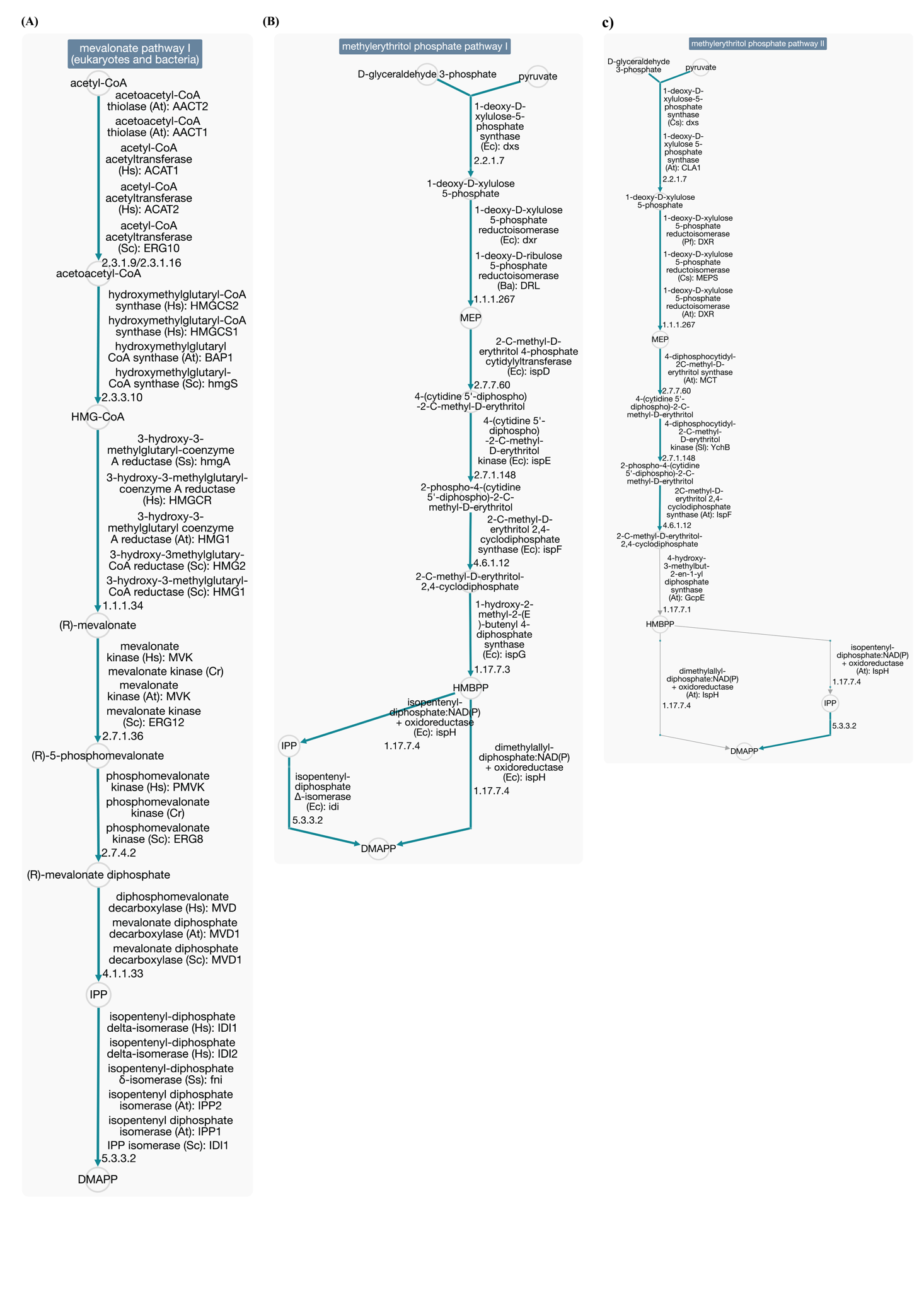


**Supplementary Figure 3.** Inflammation-related pathways related to key player isoprenoids (in particular farnesyl pyrophosphate (FPP), geranylgeranyl giphosphate (GGPP) and farnesol). Among the pathways involved in the response to inflammation, isoprenoid biosynthesis proceeds through two primary pathways: the methylerythritol phosphate (MEP) pathway (BioCyc Id: PWY-6270) and the mevalonate pathway (BioCyc Id: PWY-7391). Both pathways synthesize isopentenyl pyrophosphate (IPP) and dimethylallyl pyrophosphate (DMAPP), precursors to isoprenoid synthesis. The mevalonate pathway condenses three acetyl-CoA molecules, while the MEP pathway condenses pyruvate and D-glyceraldehyde 3-phosphate. In MetaCyc, these are further detailed as the non-mevalonate and mevalonate pathways. Subsequent synthesis of geranylgeranyl diphosphate (GGPP) and farnesol occurs via geranylgeranyl diphosphate biosynthesis (BioCyc Id: PWY-5120) and all-trans-farnesol biosynthesis (BioCyc Id: PWY-6859). MetaCyc also documents these processes as the superpathways of geranylgeranyl diphosphate biosynthesis I (via mevalonate) (BioCyc Id: PWY-5910) and II (via MEP) (BioCyc Id: PWY-5121). (A). mevalonate pathway I (eukaryotes and bacteria). (B). methylerythritol phosphate pathway I. (C). methylerythritol phosphate pathway II.
